# Linear Matrix Inequalities for an Iterative Solution of Robust Output Feedback Control of Systems with Bounded and Stochastic Uncertainty

**DOI:** 10.3390/s21093285

**Published:** 2021-05-10

**Authors:** Andreas Rauh, Swantje Romig

**Affiliations:** 1 ENSTA Bretagne, Lab-STICC, 29806 Brest, France; 2 Chair of Turbomachinery, University of Rostock, D-18059 Rostock, Germany; Swantje.Romig@uni-rostock.de

**Keywords:** robust linear matrix inequalities, uncertainty descriptions, estimation and filtering, output feedback control, stochastic optimal control problems, linear control

## Abstract

Linear matrix inequalities (LMIs) have gained much importance in recent years for the design of robust controllers for linear dynamic systems, for the design of state observers, as well as for the optimization of both. Typical performance criteria that are considered in these cases are either $H_{2}$ or $H_{\infty }$ measures. In addition to bounded parameter uncertainty, included in the LMI-based design by means of polytopic uncertainty representations, the recent work of the authors showed that state observers can be optimized with the help of LMIs so that their error dynamics become insensitive against stochastic noise. However, the joint optimization of the parameters of the output feedback controllers of a proportional-differentiating type with a simultaneous optimization of linear output filters for smoothening measurements and for their numeric differentiation has not yet been considered. This is challenging due to the fact that the joint consideration of both types of uncertainties, as well as the combined control and filter optimization lead to a problem that is constrained by nonlinear matrix inequalities. In the current paper, a novel iterative LMI-based procedure is presented for the solution of this optimization task. Finally, an illustrating example is presented to compare the new parameterization scheme for the output feedback controller—which was jointly optimized with a linear derivative estimator—with a heuristically tuned D-type control law of previous work that was implemented with the help of an optimized full-order state observer.

## 1. Introduction

One of the most important advantages of the use of LMIs in the design of robust controllers and state observers is their capability to account for bounded parameter uncertainty by means of suitable (often polytopic) uncertainty models. In such a way, it becomes possible to include a guaranteed stability proof of the uncertain linear dynamic system directly in the design stage. Moreover, polytopic uncertainty models can also be employed to over-bound the influence of nonlinear state dependencies in the system and output equations if they can be reformulated in terms of a quasi-linear representation. In addition to the task of system stabilization, further optimizations of the closed-loop dynamics become possible, which include a reduction of sensitivity against external disturbances (commonly in an $H_{\infty }$ sense) or the specification of admissible eigenvalue domains (so-called regions of Γ-stability, which serve among others as a representation for minimum damping ratios or bandwidth limitations). For general references about the theory and possible applications of LMIs in the frame of control and observer synthesis, the reader is referred to the works of [1,2,3,4,5,6,7,8]. In addition, approaches for the assignment of admissible eigenvalue domains, partially with applications to the control and oscillation attenuation of mechanical systems with elastic spring elements, were considered recently in [9,10], where a continuous-time setting was taken into account. For the discrete-time counterpart, cf. [11,12]. In addition to continuous-time systems with an integer-order time derivative, LMI techniques have recently also become an active topic of research if robust controllers and observers are to be designed for fractional-order systems with uncertainty [13,14,15,16].

As far as output feedback control procedures (instead of full state feedback controllers) are investigated in this framework, especially the work of Chesi [17] should be mentioned. However, in contrast to our paper, it did not deal with a simultaneous consideration of bounded parameter uncertainty, on the one hand, and stochastic input, system, and measurement noise, on the other hand.

Besides the design of controllers with constant, state-independent gains, LMI design procedures were also developed in recent years to allow for robust gain adaptation schemes. Moreover, an example, where methods form the field of interval analysis [18,19] were employed for an underlying reachability analysis, was published in [20]. Note that one of the attractive properties of LMIs is the existence of powerful software libraries that can be employed for a large variety of design tasks. In the current paper, the numerical implementation of the suggested LMI-based solution procedure makes use of Yalmip [21] as the user interface to MATLAB, while SeDuMi [22] is employed as the underlying solver.

In addition to the aforementioned bounded uncertainties, most practical systems are also influenced by stochastic actuator, process, and sensor noise. Assuming linear dynamics, the combination of a linear quadratic regulator design with an optimal state estimation by means of Kalman filters (for additive Gaussian noise processes) can be seen as the best solution if a feedback of all state variables is desired. However, classical formulations for the solution of this design task assume perfectly known system, input, and output matrices [23,24,25]. This aspect directly motivates the goal to employ robust LMI-based design approaches (which are naturally suited for bounded uncertainty) also in a stochastic setting. An example, where such a kind of approach was developed, was published recently by [6]. There, the problem of control parameterizations was solved such that the output and input covariances (i.e., uncertainty on the closed-loop controlled states and actuator signals) fell below specific threshold values.

A similar idea was used in [26], where LMI formulations were employed, on the one hand, to characterize the size of the domain around the system’s equilibrium for which no stability properties in the sense of a guaranteed convergence of trajectories can be made. This analysis interfaces LMIs with the Itô differential operator [27,28], which provides the possibility to define time derivatives of Lyapunov function candidates despite stochastic noise. On the other hand, the work of [26] also introduced an LMI-based numerical optimization of the gains of a full-scale state observer so that the non-provable stability domains were minimized. However, in contrast with the current paper, the approach in [26] did not consider bounded parameter uncertainty during the minimization of the domain for which stability cannot be proven. In addition, the work [26] also assumed a predefined control parameterization. Both restrictions are removed in the current paper, so that the joint optimization of output feedback controllers and linear filters can be carried out. These linear filters are, on the one hand, employed to reduce the influence of measurement noise and, on the other hand, to estimate a certain number of time derivatives of selected system outputs in a model-free way. The filtered outputs are required for the implementation of a stabilizing output feedback controller, which is optimized by an iterative LMI approach to become as insensitive as possible against parameter uncertainty and stochastic noise.

In Section 2 of this paper, the problem formulation is given. In addition, the results from [26] are briefly summarized and extended towards a combined optimization of the gains of output feedback controllers and underlying linear filters. Simulation results for a prototypical benchmark application from the area of oscillation attenuation for spring-mass-damper systems are presented in Section 3 before conclusions and an outlook for future work are given in Section 4.

## 2. LMI-Based Control and Filter Optimization

In this paper, dynamic system models are considered, which are given by the stochastic differential equations

(1) $$dx\left(t\right)=A\left(p\right)\cdot x\left(t\right)dt+B\left(p\right)\cdot \left(u\left(t\right)dt+G_{u}\left(p\right)\cdot dw_{u}\right)+G_{p}\left(p\right)\cdot dw_{p}$$

with the state vector $x:=x\left(t\right)\in R^{n_{x}}$ and the input vector $u:=u\left(t\right)\in R^{n_{u}}$; $A\left(p\right)$ and $B\left(p\right)$ are the system and input matrices, where $p\in R^{n_{p}}$ is a vector of either constant or time-varying bounded parameters. Alternatively, this vector represents the dependencies of all system matrices on the state variables x; cf. [20]. For the sake of compactness, we assume that all entries of p are mutually independent and that they influence the matrices A and B in an affine manner. Moreover, $w_{u}\in R^{m_{u}}$ and $w_{p}\in R^{m_{p}}$ are stochastically independent standard normally distributed Brownian motions of the actuator and process noise, so that $G_{u}\left(p\right)$ and $G_{p}\left(p\right)$ define the respective noise standard deviations in terms of element-wise non-negative matrix entries.

In addition, the measured system output is given by

(2) $$y_{m}\left(t\right)=C_{m}x\left(t\right)+G_{m}w_{m}\;,$$

where the output matrix $C_{m}\in R^{n_{y}\times n_{x}}$ is assumed to be exactly known; $w_{m}$ is the standard normally distributed measurement noise, while $G_{m}$ is the corresponding weighting matrix denoting the actual standard deviation of the output disturbance.

For the sake of completeness, we summarize three different control scenarios in the following, where Cases 1 and 2 are based on the implementation of state observers, while Case 3 is the linear filter-based output feedback control investigated in this paper. Note that the Cases 1 and 2 were studied in [29].

**Case** **1:** The control signal is defined as
  (3) $$u=u_{\mathrm{ff}}-K\cdot \hat{x}\;,$$
  where $u_{\mathrm{ff}}$ is a feedforward signal and $\hat{x}$ is the state estimate determined by the robust observer
  (4) $$\dot{\hat{x}}=A_{\mathrm{nom}}\cdot \hat{x}+B_{\mathrm{nom}}\cdot u+H\cdot \left(y_{m}-\hat{y}\right)$$
  that makes use of the nominal system and input matrices $A_{\mathrm{nom}}$ and $B_{\mathrm{nom}}$; see [30].
**Case** **2:** The control signal is defined as
  (5) $$u=u_{\mathrm{ff}}-K_{o}\cdot C\cdot \hat{x}\;,$$
  with the same observer as in (4).
**Case** **3:** The control signal is given by
  (6) $$u=u_{\mathrm{ff}}-K_{y}\cdot C_{y}\cdot {\hat{y}}_{f}\;,$$
  where ${\hat{y}}_{f}$ is a vector consisting of filtered system outputs and estimated output derivatives, where the filter input corresponds to the measured system outputs $y_{m}$ according to (2). Here, the negative sign in front to the controller gain matrix $K_{y}$ is introduced to make the equations structurally equivalent to the classical full-state feedback control synthesis in [29]. Moreover, without loss of generality, we assume $u_{\mathrm{ff}}=0$, which corresponds to the origin of the state space as the desired operating point.
  For what follows, we assume further that the filtered system outputs are related to selected components of an estimated state vector $\hat{x}$ by the algebraic relation
  (7) $$\bar{C}\cdot \hat{x}=C_{y}\cdot {\hat{y}}_{f}\;.$$
  The introduction of this constraint allows us to formulate stability requirements for an output feedback control—that is based on an ideal filtering—(i.e., the algebraic relation (7) holds) in terms of matrix inequalities, which can be cast into LMIs by a suitable change of coordinates; see Section 2.2. Errors, which inevitably result from the non-negligible filter dynamics, are later on taken into consideration in Section 2.3 and Section 2.4, especially in Equations (28) and (29).
  If the matrix $C_{y}$ (which extracts certain state variables or their linear combinations from the linear filter’s state vectors) has full row rank, (7) can be reformulated according to
  (8) $${\hat{y}}_{f}=C\cdot \hat{x}\;\mathrm{with}\;C=C_{y}^{+}\cdot \bar{C}\;,$$
  where
  (9) $$C_{y}^{+}={\left(C_{y}^{T}C_{y}\right)}^{-1}C_{y}^{T}$$
  is the matrix pseudo inverse. Under the assumption of the aforementioned stationary, i.e., purely algebraic, relation, the matrix C provides the possibility to express the filter outputs ${\hat{y}}_{f}$ in terms of the internal states of the plant (1).

**Figure 1:**
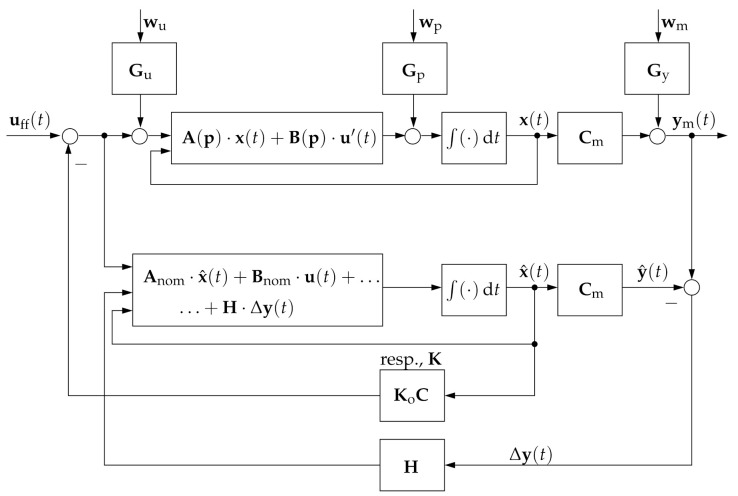
Observer-based state and output feedback control structure according to Case 1 with the gain matrix K and Case 2 with the structured gain $K_{o}C$.

**Figure 2:**
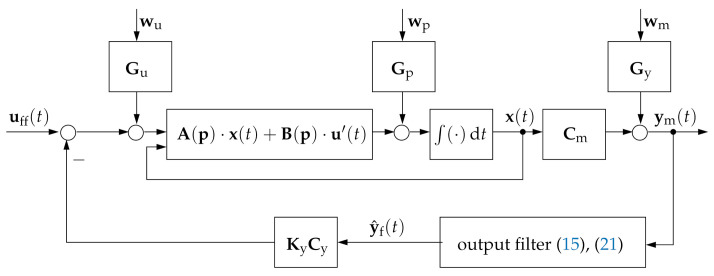
Filter-based output feedback control structure according to Case 3.

For further details concerning the structured, LMI-based output feedback control design in Case 2, the reader is also referred to [31]. Figure 1 and Figure 2 give a summary of the three different types of control structures described above, where the last one is the focus of this paper.

To guarantee the solvability of the control design task, it is assumed that the system (1) is stabilizable using either of the inputs (3), (5), or (6). In addition, the pair $\left(A\left(p\right),C\right)$ needs to be robustly observable (or at least detectable) in Cases 1 and 2; cf. [29].

### 2.1. Polytopic Uncertainty Modeling

As shown in [4,32], it is possible to describe the influence of uncertainty in many practical applications by bounded domains D of the polytope type. For that purpose, it is necessary that all system matrices in (1) belong to a convex combination of extremal vertex matrices in the form

(10) $$\begin{array}{cc} D=\{ & \left[A,B,G_{u},G_{p}\right]|\left[A\left(\xi \right),B\left(\xi \right),G_{u}\left(\xi \right),G_{p}\left(\xi \right)\right]\\ \; & =\sum_{v=1}^{n_{v}}\xi_{v}\cdot \left[A_{v},B_{v},G_{u,v},G_{p,v}\right];\sum_{v=1}^{n_{v}}\xi_{v}=1;\xi_{v}\geq 0\}\;, \end{array}$$

where $n_{v}$ denotes the number of independent extremal realizations for the union of all four matrices included in (10).

### 2.2. Robust Output Feedback Control for Case 3

LMI-based design approaches can be employed for the design of output feedback controllers that are restricted in their parameterization according to Case 3. Here, the system’s measured outputs and selected time derivatives of these signals are fed back after a suitable low-pass filtering, parameterized according to the following subsections.

In the case of an ideal (error-free) filtering, the closed-loop dynamics are guaranteed to be robustly stable if the controller gains $K_{y}$ satisfy the following theorem representing a bilinear matrix inequality.

**Theorem** **1.** *(Sufficient stability condition for robust output feedback control) Robust asymptotic stability of the closed-loop control system according to Case 3 is ensured for an error-free output feedback (i.e., $x\equiv \hat{x}$) if the gain matrix $K_{y}$ satisfies the bilinear matrix inequalities*

(11) $${\left(A_{v}-B_{v}K_{y}C_{y}^{+}\bar{C}\right)}^{T}P+P\cdot \left(A_{v}-B_{v}K_{y}C_{y}^{+}\bar{C}\right)≺0\;,$$

$P=P^{T}≻0$, *for all vertices $v\in \{1,\ldots ,n_{v}\}$ in (10)*.

**Proof.**  The proof of Theorem 1 is a direct consequence of setting up sufficient stability conditions for each vertex system of a linear model with polytopic uncertainty representation. In this way, Equation (11) represents the Lyapunov inequalities to be satisfied for each vertex system according to [8,33]. □

Note, the matrix inequality (11) is bilinear due to multiplicative couplings between the yet unknown matrices $K_{y}$ and P. The following corollary provides a possibility to transfer these stability requirements into computationally feasible LMIs including a linear equality constraint.

**Corollary** **1.** *An LMI formulation of Theorem 1 is obtained by introducing a linearizing change of variables with the positive definite, symmetric unknown matrix $Q=Q^{T}=P^{-1}≻0$, as well as the equality constraints*

(12) $$MC_{y}^{+}\bar{C}=C_{y}^{+}\bar{C}Q\;\mathrm{and}\;N=K_{y}M\;,$$

*for which $C_{y}^{+}\cdot \bar{C}$ was assumed to be precisely known, i.e., a point matrix, according to its definition in Equations (6) and (7). Substituting the relations (12) into (11) and multiplying the matrix inequality form the left and right by Q yield the LMIs*

(13) $$A_{v}Q+Q{A_{v}}^{T}-B_{v}NC_{y}^{+}\bar{C}-{\left(C_{y}^{+}\bar{C}\right)}^{T}N^{T}{B_{v}}^{T}≺0$$

*to be jointly satisfied for each vertex system $v\in \{1,\ldots ,n_{v}\}$.*

*If the matrix C has full row rank, the algebraic constraint in (12) ensures that M has full rank and that it is therefore invertible. Then, the resulting controller gain is given by [33]:*

(14) $$K_{y}=NM^{-1}\;.$$

### 2.3. Linear Output Filtering

As shown in [26], a linear low-pass output filtering, as well as the derivative estimation of the scalar measured variables $y_{m,i}$, $i\in \{1,\ldots ,n_{y}\}$, can be described in terms of the input-output representation

(15) $$\sum_{k=0}^{\xi_{i}}\alpha_{k,i}\cdot \frac{d^{k}y_{f,i}}{dt^{k}}=\alpha_{0,i}\cdot y_{m,i}\;.$$

The linear differential Equation (15) has the order $\xi_{i}$ and contains the *k*-th order time derivatives $y_{f,i}^{\left(k\right)}:=\frac{d^{k}y_{f,i}}{dt^{k}}$ that represent the filtered quantities that can be utilized in the controller according to Case 3, Equation (6). In this subsection, we present an LMI-based design of these filters as a systematic generalization of the pole (respectively, time constant) assignment that was performed in [26].

When additionally accounting for the influence of stochastic noise with quasi-continuous measurements, Equation (15) turns into the state-space representation

(16) $$dy_{f,i}=\left(\left(A_{f,i}-b_{f,i}k_{f,i}^{T}\right)\cdot y_{f,i}+\left(b_{f,i}e_{1}^{T}k_{f,i}\right)\cdot y_{m,i}^{\prime }\right)\;dt+\left(\left(b_{f,i}e_{1}^{T}k_{f,i}\right)\cdot g_{m,i}\right)\;dw_{m,i}$$

of a stochastic differential equation with the state vector

(17) $$y_{f,i}={\left[\begin{array}{ccc} y_{f,i} & \ldots & y_{f,i}^{\left(\xi_{i}-1\right)} \end{array}\right]}^{T}\;,$$

in which the superscript index denotes the corresponding temporal derivative order, the coefficient matrices

(18) $$A_{f,i}=\left[\begin{array}{ccccc} 0 & 1 & 0 & \ldots & 0\\ 0 & 0 & 1 & \ldots & 0\\ \vdots & \; & & \ddots & \vdots \\ 0 & 0 & 0 & \ldots & 1\\ 0 & 0 & 0 & \ldots & 0 \end{array}\right]\in R^{\xi_{i}\times \xi_{i}}\;,\;b_{f,i}=\left[\begin{array}{c} 0\\ 0\\ \vdots \\ 1 \end{array}\right]\in R^{\xi_{i}}\;,$$

the first unit vector $e_{1}={\left[\begin{array}{cccc} 1 & 0 & \ldots & 0 \end{array}\right]}^{T}\in R^{\xi_{i}}$, and the yet unknown filter gain vector

(19) $$k_{f,i}={\left[\begin{array}{ccc} \alpha_{0,i} & \ldots & \alpha_{\xi_{i}-1,i} \end{array}\right]}^{T}$$

with $\alpha_{\xi_{i},i}\equiv 1$. This simplification results from a normalization of both sides of (15) under the restriction of steady-state accuracy due to which the derivatives of order zero on both sides of (15) have identical coefficients. For the sake of compactness, it is assumed that the matrix $G_{y}$ in (2) is purely diagonal. This corresponds to vanishing correlations between the noise of all scalar measurements in (2) with $y_{m,i}=y_{m,i}^{\prime }+g_{m,i}w_{m,i}$.

Hence, the low-pass filtered derivative of the order *j*, $j\in \{0,\ldots ,\xi_{i}\}$, for the *i*-th measured output is related to the state vector $y_{f,i}$ of the stochastic differential equation model (16) by

(20) $$\begin{array}{c} {\hat{y}}_{f,i}^{\left(j\right)}=\left\{\begin{array}{cc} e_{j+1}^{T}y_{f,i}+0\cdot y_{m,i} & \mathrm{for}\;j\in \{0,\ldots ,\xi_{i}-1\}\\ e_{\xi_{i}}^{T}A_{f_{i}}y_{f,i}+\alpha_{0,i}\cdot \left(y_{m,i}^{\prime }+g_{m,i}w_{m,i}\right) & \mathrm{for}\;j=\xi_{i}\;, \end{array}\right. \end{array}$$

with $e_{j}\in R^{\xi_{i}}$ denoting the *j*-th unit vector. In the equations above, the subscript m denotes the measured data, the prime symbol ${\left(\cdot \right)}^{\prime }$ the ideal noise-free outputs, the subscript f the filtered data, and $\hat{\left(\cdot \right)}$ the estimates used by the controller.

A compact notation of the filtered output vector

(21) $${\hat{y}}_{f}={\left[\begin{array}{ccc} {\hat{y}}_{f,1}^{T} & \ldots & {\hat{y}}_{f,n_{y}}^{T} \end{array}\right]}^{T}$$

in Equation (6) is obtained by collecting all outputs from (20) that are actually relevant for the output feedback design according to

(22) $${\hat{y}}_{f,i}=C_{f,i}\cdot y_{f,i}+D_{f,i}\cdot \left(y_{m,i}^{\prime }+g_{m,i}w_{m,i}\right)\;.$$

Here, $C_{f,i}$ represents the dependence of the filter outputs ${\hat{y}}_{f,i}$ on the filters’ state variables $y_{f,i}$ and contains the coefficients of the first summand of both rows in (20). The factor $D_{f,i}$ is only non-zero if the filter has a direct measurement feedthrough (and, thus, also a noise feedthrough) because the approximate of the derivative of the order $\xi_{i}$ is expressed in terms of the last vector component of the dynamic model (16).

The asymptotic stability of the filter dynamics with purely real eigenvalues is ensured by the following theorem.

**Theorem** **2.** *(Asymptotically stable, non-oscillatory filter dynamics) The filter dynamics (20) are guaranteed to be asymptotically stable with purely real eigenvalues of the deterministic part of the stochastic differential Equation (16), if the gain vectors $k_{f,i}$ satisfy the matrix inequalities*

(23) $$D_{0}⊗P_{f,i}+D_{1}⊗\left(\left(A_{f,i}-b_{f,i}k_{f,i}^{T}\right)\cdot P_{f,i}\right)+D_{1}^{T}⊗{\left(\left(A_{f,i}-b_{f,i}k_{f,i}^{T}\right)\cdot P_{f,i}\right)}^{T}⪯0$$

*with some $P_{f,i}=P_{f,i}^{T}≻0$ for all $i\in \{1,\ldots ,n_{y}\}$, where*

(24) $$D_{0}=\left[\begin{array}{cccc} 2\gamma & 0 & 0 & 0\\ 0 & -2\delta & 0 & 0\\ 0 & 0 & 0 & 0\\ 0 & 0 & 0 & 0 \end{array}\right]\;,\;\;D_{1}=\left[\begin{array}{cccc} 1 & 0 & 0 & 0\\ 0 & -1 & 0 & 0\\ 0 & 0 & \sin\theta & \cos\theta \\ 0 & 0 & -\cos\theta & \sin\theta \end{array}\right]$$

*with $0\leq \theta <\frac{\pi }{2}$; ⊗ is the matrix Kronecker product of the respective arguments; γ>0 and δ>γ represent bounds on the real parts of the eigenvalues $s_{i}$ so that $-\delta \leq ℜ\{s_{i}\}\leq -\gamma$ holds. To obtain purely real eigenvalues, θ=0 is chosen.*

**Figure 3:**
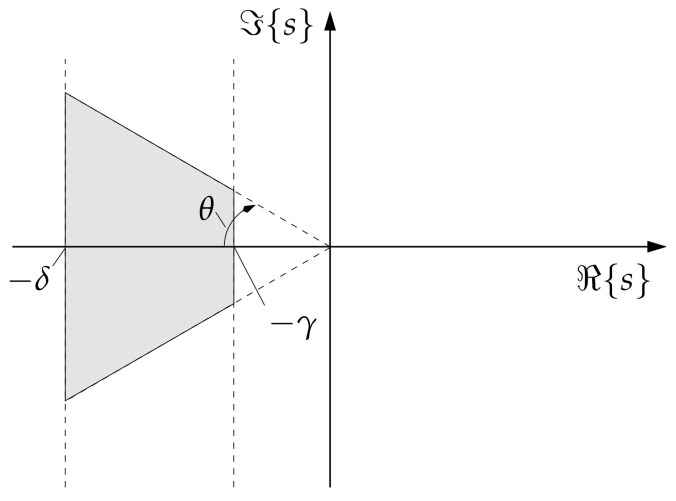
Domain of the eigenvalues compatible with the constraints (23) and (24), where θ=0 is desired to guarantee non-oscillatory dynamics.

A graphical representation of the stability domain represented by (23) with (24) is given in Figure 3.

**Proof.**  Theorem 2 is a direct consequence of formulating a bounded interval $\left[-\delta ;\;-\gamma \right]$ on the negative real axis of the complex *s* plane (with $\bar{s}$ being the conjugate complex of *s*) as the desired Γ-stability domain

(25) $$F_{\Gamma }\left(s\right):=D_{0}+s\cdot D_{1}+\bar{s}\cdot D_{1}^{T}⪯0$$

according to [3,5,7]. For a detailed derivation of the coefficient matrices $D_{0}$ and $D_{1}$, see Appendix A. A reformulation of this Γ-stability domain into a gain-dependent matrix inequality according to ([20], Equation (11)) completes the proof. □

**Remark** **1.** *The specification of* Γ*-stability domains is analogously possible for the output feedback parameterization. For a corresponding generalized formulation, see Appendix B. From a practical point of view, enforcing purely real eigenvalues with θ=0 in the filter parameterization is often not necessary. Commonly, it is sufficient to specify large enough damping ratios, for example from the sector $0\leq \theta <\frac{\pi }{4}$, where the upper bound of this interval would correspond to the value $\frac{1}{2}\sqrt{2}$ for Lehr’s damping coefficient in a second-order differential equation.*

**Corollary** **2.** *Following the linearizing change of variables*

(26) $$P_{f,i}=Q_{f,i}^{-1}\;and\;k_{f,i}^{T}=\phi_{f,i}^{T}P_{f,i}$$

*and multiplying (23) from the left and right with the matrix $I⊗Q_{f,i}$, $I\in R^{4\times 4}$, $Q_{f,i}=Q_{f,i}^{T}≻0$ lead to the equivalent LMIs*

(27) $$D_{0}⊗Q_{f,i}+D_{1}⊗\left(Q_{f,i}A_{f,i}^{T}-\phi_{f,i}b_{f,i}^{T}\right)+D_{1}^{T}⊗\left(A_{f,i}Q_{f,i}-b_{f,i}\phi_{f,i}^{T}\right)≺0\;.$$

### 2.4. Optimal Output Feedback Control

Under the consideration of the structure of the control law of Case 3, the stochastic differential Equation (1) for the controlled polytopic system model turns into

(28) $$dx_{v}=\left(\left(A_{v}-B_{v}K_{y}C_{y}^{+}\bar{C}\right)x_{v}+B_{v}K_{y}C_{y}e_{f}\right)dt+\left[\begin{array}{cc} B_{v}G_{u,v} & G_{p,v} \end{array}\right]\;\cdot \;\left[\begin{array}{c} dw_{u}\\ dw_{p} \end{array}\right]\;.$$

In addition, the ideal filtering process (assuming a noise-free setting, where the following equation turns exactly into a disturbance-free ordinary differential equation representation in which ${\bar{y}}_{f,i}$ represents the state vector after removing the noise term from (16)) is described by

(29) $$d{\bar{y}}_{f,i}=\left(\left(A_{f,i}-b_{f,i}k_{f,i}^{T}\right)\cdot {\bar{y}}_{f,i}+\left(b_{f,i}e_{1}^{T}k_{f,i}\right)\cdot y_{m,i}^{\prime }\right)\;dt\;.$$

After introducing the vectors of output estimation errors

(30) $$e_{f,i}={\bar{y}}_{f,i}-y_{f,i}\;,\;\;i\in \left\{1,\ldots ,n_{y}\right\}\;,$$

and a stacked vector notation $e_{f}$ according to (21), the error dynamics of the linear filters are given by

(31) $$de_{f,i}=\left(\left(A_{f,i}-b_{f,i}k_{f,i}^{T}\right)\cdot e_{f,i}\right)\;dt-\left(\left(b_{f,i}e_{1}^{T}k_{f,i}\right)\cdot g_{m,i}\right)\;dw_{m,i}\;.$$

Now, introduce the stacked vector

(32) $$z_{v}={\left[\begin{array}{cccc} x_{v}^{T} & e_{f,i}^{T} & \ldots & e_{f,n_{y}}^{T} \end{array}\right]}^{T}$$

consisting of system states and noise-induced filter errors. The stochastic differential equations corresponding to (32) are given by

(33) $$dz_{v}=A_{v}\cdot z_{v}\;dt+G_{v}\cdot \left[\begin{array}{c} dw_{u}\\ dw_{p}\\ dw_{m} \end{array}\right]$$

with the system matrix

(34) $$A_{v}=\left[\begin{array}{cc} A_{v}-B_{v}K_{y}C_{y}^{+}\bar{C} & B_{v}K_{y}C_{y}\\ 0 & A_{v,2,2} \end{array}\right]\;,$$

in which its lower right sub-block has the block diagonal structure

(35) $$A_{v,2,2}=\mathrm{blkdiag}\left(\left(A_{f,1}-b_{f,1}k_{f,1}^{T}\right),\ldots ,\left(A_{f,n_{y}}-b_{f,n_{y}}k_{f,n_{y}}^{T}\right)\right)$$

and the matrix of standard deviations

(36) $$G_{v}=\left[\begin{array}{ccc} B_{v}G_{u,v} & G_{p,v} & 0\\ 0 & 0 & G_{v,2,3} \end{array}\right]\;,$$

with the block diagonal sub-matrix

(37) $$G_{v,2,3}=-\mathrm{blkdiag}\left(\left(b_{f,1}e_{1}^{T}k_{f,i}\right)\cdot g_{m,1},\ldots ,\left(b_{f,n_{y}}e_{1}^{T}k_{f,n_{y}}\right)\cdot g_{m,n_{y}}\right)\;.$$

**Theorem** **3.** *(Optimal control and filter gains) The controller and filter gains from Corollary 1 in Section 2.2 and Corollary 2 in Section 2.3 are jointly optimal if they are chosen so that the cost function*

(38) $$J=\sum_{v=1}^{n_{v}}\left(\frac{\mathrm{trace}\left\{N_{v}\right\}}{\det\left(-{\overset{ˇ}{\bar{A}}}_{v}\right)}\cdot \frac{1}{\det\left(\overset{ˇ}{Q}\right)\cdot \prod_{i=1}^{n_{y}}\det\left({\overset{ˇ}{Q}}_{f,i}\right)}\right)$$

*is minimized, where the abbreviation ${\overset{ˇ}{\bar{A}}}_{v}={\overset{ˇ}{A}}_{v}^{T}P+P{\overset{ˇ}{A}}_{v}≺0$ is defined and $N_{v}=N_{v}^{T}≻0$ is a free matrix variable. Here, the matrices ${\overset{ˇ}{A}}_{v}$ are defined for the vertices of the polytope (10) according to*

(39) $${\overset{ˇ}{A}}_{v}=\left[\begin{array}{cc} A_{v}-B_{v}{\overset{ˇ}{K}}_{y}C_{y}^{+}\bar{C} & B_{v}{\overset{ˇ}{K}}_{y}C_{y}\\ 0 & {\overset{ˇ}{A}}_{v,2,2} \end{array}\right]$$

*with*

(40) $${\overset{ˇ}{A}}_{v,2,2}=\mathrm{blkdiag}\left(\left(A_{f,1}-b_{f,1}{\overset{ˇ}{k}}_{f,1}^{T}\right),\ldots ,\left(A_{f,n_{y}}-b_{f,n_{y}}{\overset{ˇ}{k}}_{f,n_{y}}^{T}\right)\right)\;.$$

*In addition, the definiteness constraint*

(41) $$\left[\begin{array}{cc} N_{v} & {\overset{ˇ}{G}}_{v}^{T}\\ {\overset{ˇ}{G}}_{v} & \left[\begin{array}{cc} Q & 0\\ 0 & \mathrm{blkdiag}\left(Q_{f,1},\ldots ,Q_{f,n_{y}}\right) \end{array}\right] \end{array}\right]≻0$$

*with*

(42) $${\overset{ˇ}{G}}_{v}=\left[\begin{array}{ccc} B_{v}G_{u,v} & G_{p,v} & 0\\ 0 & 0 & {\overset{ˇ}{G}}_{v,2,3} \end{array}\right]$$

*and*

(43) $${\overset{ˇ}{G}}_{v,2,3}=-\mathrm{blkdiag}\left(\left(b_{f,1}e_{1}^{T}{\overset{ˇ}{k}}_{f,i}\right)\cdot g_{m,1},\ldots ,\left(b_{f,n_{y}}e_{1}^{T}{\overset{ˇ}{k}}_{f,n_{y}}\right)\cdot g_{m,n_{y}}\right)$$

*must be satisfied; $\overset{ˇ}{\left(\cdot \right)}$ symbols indicate an iterative evaluation, where all such values are replaced by the outcome of the previous iteration stage.*

**Proof.**  Define a positive definite Lyapunov function candidate

(44) $$V\left(z_{v}\right)=\frac{1}{2}\left(z_{v}^{T}Pz_{v}\right)$$

with the block diagonal matrix

(45) $$P=\mathrm{blkdiag}\left(P,P_{f,1},\ldots ,P_{f,n_{y}}\right)\;.$$

By applying the Itô differential operator [27], its time derivative is obtained as

(46) $$L\left(V\right)=\frac{1}{2}\left(z_{v}^{T}\left(A_{v}^{T}P+PA_{v}\right)z_{v}+\mathrm{trace}\left\{G_{v}^{T}PG_{v}\right\}\right)\;.$$

Following the reasoning in [26], the interior of the ellipsoid

(47) $$z_{v}^{T}M_{v}^{-1}z_{v}-1=0\;,$$

where

(48) $$M_{v}^{-1}=\left(\frac{-{\bar{A}}_{v}}{\mathrm{trace}\left\{G_{v}^{T}PG_{v}\right\}}\right)$$

and

(49) $${\bar{A}}_{v}:=A_{v}^{T}P+PA_{v}≺0$$

hold, is the domain for which no stability properties can be verified. Its volume is proportional to

(50) $$\sqrt{\det\left(M_{v}\right)}=\sqrt{\frac{\mathrm{trace}\left\{G_{v}^{T}PG_{v}\right\}}{\det\left(-{\bar{A}}_{v}\right)}}\;.$$

Generalizing the statements from [26], the minimization of the ellipsoid volume—with a simultaneous maximization of the error domain for which the linear feedback signals are bounded by some positive constant according to [34] after introducing the denominator terms depending on Q and $Q_{f,i}$—leads to the cost

(51) $$J_{v}=\frac{\mathrm{trace}\left\{G_{v}^{T}PG_{v}\right\}}{\det\left(-{\bar{A}}_{v}\right)}\cdot \frac{1}{\det\left(Q\right)\cdot \prod_{i=1}^{n_{y}}\det\left(Q_{f,i}\right)}$$

to be minimized for each vertex *v*. Nonlinearities in the argument $G_{v}^{T}PG_{v}$ of the trace in (51) are removed by a relaxation into the matrix inequality

(52) $$N_{v}≻G_{v}^{T}PG_{v}\;\;\mathrm{corresponding}\;\mathrm{to}\text{:}\;\;N_{v}-G_{v}^{T}PG_{v}≻0$$

with $N_{v}=N_{v}^{T}≻0$, which finally leads to

(53) $$\left[\begin{array}{cc} N_{v} & G_{v}^{T}\\ G_{v} & \left[\begin{array}{cc} Q & 0\\ 0 & \mathrm{blkdiag}\left(Q_{f,1},\ldots ,Q_{f,n_{y}}\right) \end{array}\right] \end{array}\right]≻0$$

by applying the Schur complement formula. Summing up the expressions (51) for all $v\in \{1,\ldots ,n_{y}\}$, as well as replacing the denominator terms depending on the gain values in (34) by their result from the previous iteration step and doing the same with the gains in (53) complete the proof. □

**Figure 4:**
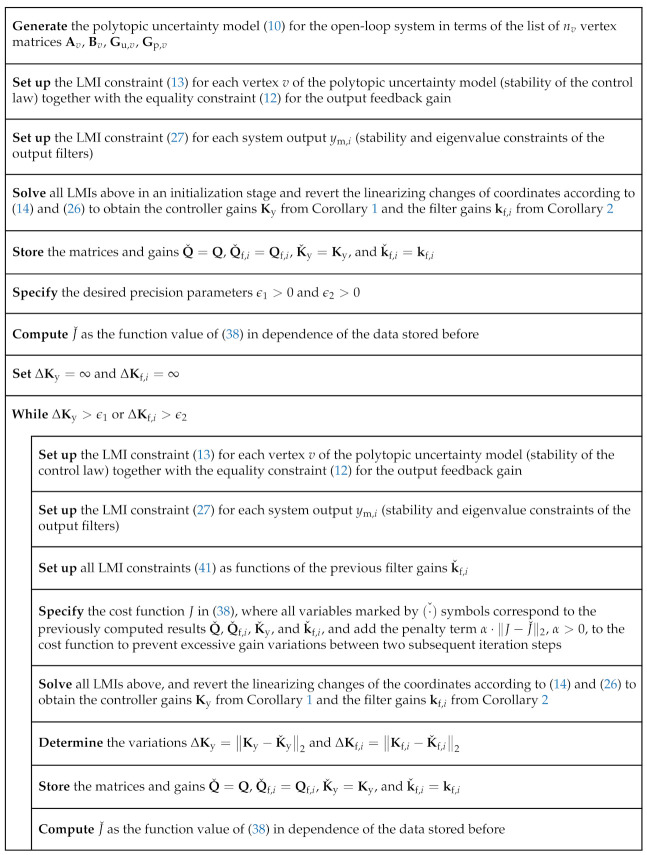
Structure diagram of the iteration procedure for the proposed filter-based control parameterization.

Figure 4 provides a structure diagram of the complete iteration process for the parameterization of the filter-based control law of Case 3. There, the precision parameters $\epsilon_{1}>0$ and $\epsilon_{2}>0$ need to be chosen so that they are much smaller than the norms of the gains $K_{y}$ and $k_{f,i}$ resulting from the initialization phase that is carried out prior to the while-loop, for example $\epsilon_{1}=10^{-6}\cdot {∥K_{y}∥}_{2}$ and $\epsilon_{2}=10^{-6}\cdot {∥k_{f,i}∥}_{2}$.

**Remark** **2.** *For the examples considered in the following section, the while-loop typically terminated after no more than 30 iterations, where each iteration step took less than a second on a standard notebook computer.*

## 3. Simulation Results

To demonstrate the suggested solution procedure, the oscillation attenuation of a spring-mass-damper system with the position variable $x_{1}$, the velocity $x_{2}$, and the actuating force $x_{3}$ is considered. It is described by the state equations

(54) $$dx=\left(\left[\begin{array}{ccc} 0 & 1 & 0\\ a_{21} & a_{22} & a_{23}\\ 0 & 0 & a_{33} \end{array}\right]x+\left[\begin{array}{c} 0\\ 0\\ b_{3} \end{array}\right]u\right)dt+g_{p}\;dw_{p}$$

with the nominal system parameters $a_{21}=a_{21,\mathrm{nom}}=-200$, $a_{22}=a_{22,\mathrm{nom}}=-15$, $a_{23}=a_{23,\mathrm{nom}}=-400$, $a_{33}=a_{33,\mathrm{nom}}=-200$, $b_{3}=b_{3,\mathrm{nom}}=10$, and $g_{p}={\left[\begin{array}{ccc} 0 & 0.1 & 0 \end{array}\right]}^{T}$.

Stochastic input disturbances $g_{u}$ are neglected in this example. The third state equation in (54) describes the input force $x_{3}$ that is generated from the control signal *u* by a first-order lag element with the time constant $|a_{33}{|}^{-1}$.

Noisy measurements of the position are available according to

(55) $$y_{m}=x_{1}+g_{m}\;w_{m}$$

with the standard deviation $g_{m}=0.5$.

### 3.1. Control Design for the Nominal System Model with Precisely Known Parameters

To perform the oscillation attenuation, a differentiating control law is implemented in terms of a feedback of an approximation of the velocity $x_{2}$ by means of $u=-K_{D}{\hat{x}}_{2}$ with a suitably chosen, stabilizing gain value $K_{D}\in R$.

Setting

**Figure 5:**
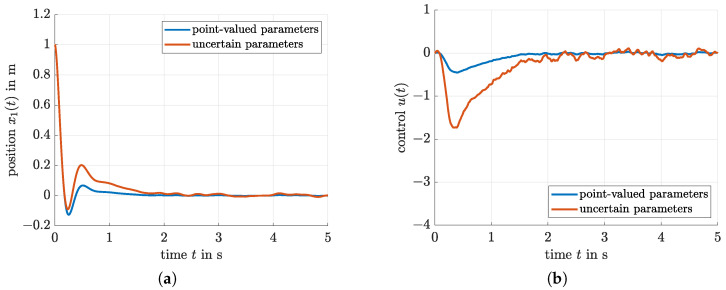
Control performance of the proposed iterative LMI-based optimization technique. (**a**) Position $x_{1}$ for the spring-mass-damper system. (**b**) Control signal *u* for the spring-mass-damper system.

(56) γ=2.5andδ=50

for the range of admissible eigenvalues in Theorem 2, the gain

(57) $$K_{D}\approx -0.386$$

with

(58) $$k_{f}^{T}\approx \left[\begin{array}{ccc} 578.4 & 356.2 & 53.7 \end{array}\right]$$

is obtained with ξ=3 if the algorithm summarized in Figure 4 is applied. Corresponding simulation results for the controlled position $x_{1}$ and the system input *u* are shown in Figure 5a,b. These graphs further contain a comparison with the simulation results for the control and filter optimization when the polytopic system model described in the following subsection is considered.

### 3.2. Control Design for a Polytopic System Model

If it is assumed in a robust control design that $a_{21}$ and $a_{22}$ can vary independently in the intervals $a_{21}\in a_{21,\mathrm{nom}}\cdot \left[0.5;\;1.5\right]$ and $a_{22}\in a_{22,\mathrm{nom}}\cdot \left[0.5;\;1.5\right]$, while all remaining parameters are set equal to the previous point values, the control and filter gains obtained from the the algorithm in Figure 4 change to

(59) $$K_{D}\approx -1.56$$

with

(60) $$k_{f}^{T}\approx \left[\begin{array}{ccc} 589.9 & 357.9 & 52.4 \end{array}\right].$$

**Figure 6:**
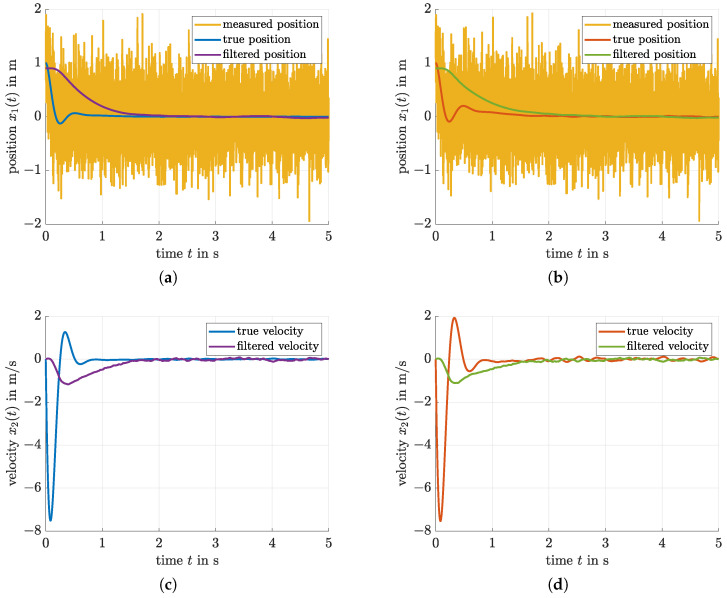
Reconstruction of the states $x_{1}$ and $x_{2}$ for the proposed iterative LMI-based filter and control optimization. (**a**) Reconstruction of the position $x_{1}$ in comparison with the noisy measurement and the true state evolution (setting from Section 3.1). (**b**) Reconstruction of the position $x_{1}$ in comparison with the noisy measurement and the true state evolution (setting from Section 3.2). (**c**) Reconstruction of the velocity $x_{2}$ in comparison with the true state evolution (setting from Section 3.1). (**d**) Reconstruction of the velocity $x_{2}$ in comparison with the true state evolution (setting from Section 3.2).

Also in this case (Figure 5a,b), an efficient oscillation attenuation is obtained, where the simulation was carried out for the nominal system parameters. In addition, Figure 6 provides a comparison of the true and estimated states $x_{1}$ and $x_{2}$ for the model-free filter technique that was optimized by means of the proposed LMI-based procedure. On the one hand, it can be seen that the resulting parameterization is capable of effectively suppressing the stochastic measurement noise. However, in contrast to the observer discussed in the following subsection, the price to pay for this noise suppression is a non-negligible delay in the reconstruction of both $x_{1}$ and $x_{2}$.

### 3.3. Comparison with a Heuristic D-Type Control Parameterization

**Figure 7:**
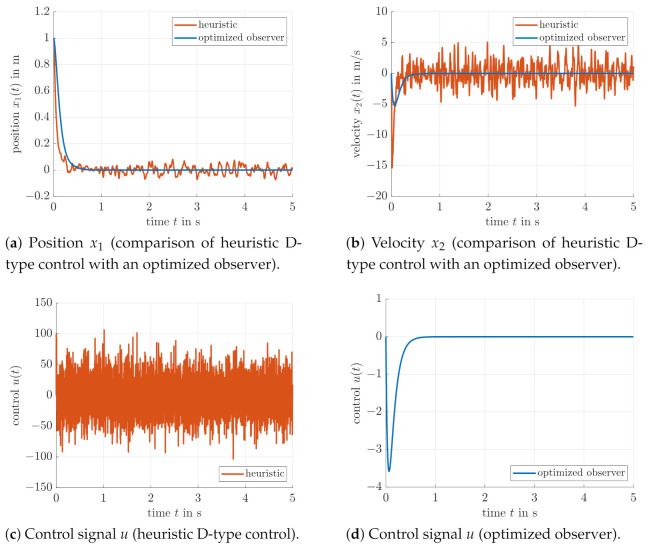
Comparison of a heuristic D-type control approach with an optimized observer from the previous work [26].

**Figure 8:**
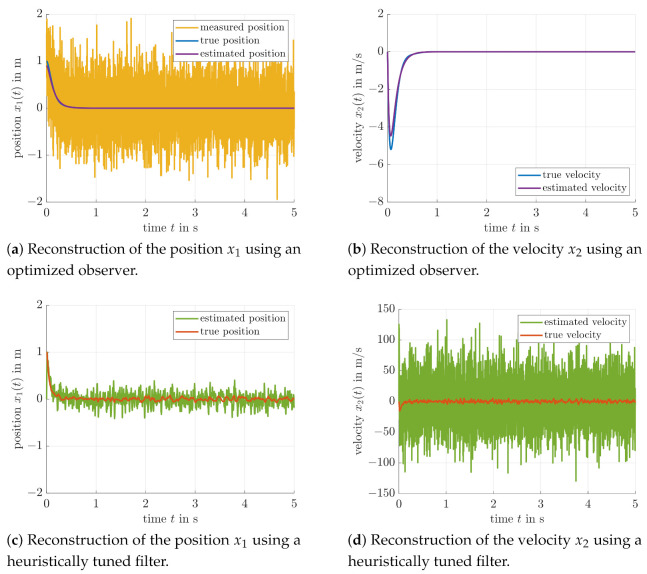
Comparison of a heuristic D-type control approach with an optimized observer from the previous work [26] (cont’d).

For the sake of comparison, Figure 7 and Figure 8 contain the results of the heuristically tuned control approach from [26], where a root locus analysis of the plant was employed to set the controller gain to $K_{D}=-0.8$ to obtain purely negative real eigenvalues. If the low-pass filtered velocity estimate is determined by a second-order transfer function with the time constants $T_{1}={\left(2\pi \cdot 32\right)}^{-1}$ and $T_{2}=0.5T_{1}$, excessively large control inputs can be observed, which are more or less useless in practice due to extreme actuator wear and energy consumption.

Although this was not discussed explicitly in this paper, it is easily possible to extend the newly derived design LMIs of the output feedback according to Corollary 1 by further requirements. Especially, Γ-stability domains can be introduced not only to enforce real filter eigenvalues, but also to guarantee desired transition times and bandwidth limitations of the controller itself. The required steps are summarized in Appendix B.

For a second comparison with [26], Figure 7 and Figure 8 also contain a further velocity estimation approach. There, the same (heuristically chosen) gain $K_{D}=-0.8$ was used for the controller parameterization; however, an LMI-based observer tuning was performed on the basis of a nominal system model. The corresponding results are well comparable with the more simple filter-based output feedback from this paper with respect to noise suppression and transient behavior of the controlled system. Obviously, however, the use of a full-scale state observer leads to a suppression of undershooting the desired target position $x_{1}=0$ due to the fact that the velocity estimates are less affected by the lag behavior that occurs inevitably in the case of a model-free linear filter approach for derivative estimation. This becomes obvious if the Figure 6c,d are compared with Figure 8b. However, the heuristically parameterized second-order filter-based velocity estimate in Figure 8d is by far worse than the optimized filter in the Figure 6c,d and the model-based observer in Figure 8b.

Therefore, it should be pointed out that using the joint optimization of filter-based derivative estimators and output feedback controller gains is especially promising in practice if either a purely proportional feedback is implemented or if the use of no more than two time derivatives of the measured signals is required. In other scenarios, the Cases 1 and 2 sketched in this paper (cf. [29] for further details) are superior in transient operating conditions due to the capability of a full-scale state observer to reduce not only the effect of stochastic noise, but also to avoid large undesired lag phenomena.

## 4. Conclusions and Outlook on Future Work

In this paper, a novel approach for the combined optimization of output feedback controller gains and linear filter transfer functions was proposed for linear continuous-time dynamic systems. This approach took into account stochastic disturbances in both the system dynamics and measurement model and aimed at finding parameterizations with which the domains around the system’s equilibrium, for which stability cannot be proven in a stochastic sense, are minimized. Due to the use of an LMI-based formulation of the optimization task, it is easily applicable to systems with bounded parameter uncertainty.

Future work will aim at validating the proposed design methodology experimentally and at interfacing it with LMI-based design approaches for interval observers [35] as a technique for the state estimation in a bounded-error framework. In addition, also combinations with sliding mode-type control procedures such as those in [36] can be investigated. Finally, it should be pointed out that the technique is readily applicable also to higher dimensional system models, such as the interconnection of multiple spring-mass-damper elements in the frame of mechanical vibration control or the interconnection of RLC networks, which may serve either as a representation of long electric transmission lines or as a finite-dimensional approximation of volume flow and pressure variations in fluidic networks [10,37,38]. In all of these applications, efficient output feedback control procedures are promising for the reduction of undesirable oscillations. However, future work should not only apply the proposed methodology to systems where the measured quantities are already predefined. Instead, novel optimization procedures for the most effective sensor placement should be developed and combined with the approach presented in this paper.

## Data Availability

The data are contained within the article.

## Appendix A. Derivation of the Matrices D 0 and D 1 in (24)

This Appendix A provides an element-by-element derivation of the matrices $D_{0}$ and $D_{1}$ in (24). The first constraint (right bound for the eigenvalues’ real part) on the area of admissible eigenvalues is given by

(A1) ℜ{s}≤−γ.

It is equivalent to

(A2) $$\frac{1}{2}\cdot \left(s+\bar{s}\right)\leq -\gamma \;⟹\;2\gamma +s+\bar{s}\leq 0\;.$$

Analogously, the second constraint (left bound for the eigenvalues’ real part) is given by

(A3) ℜ{s}≥−δ.

It is equivalent to

(A4) $$\frac{1}{2}\cdot \left(s+\bar{s}\right)\geq -\delta \;⟹\;2\delta +s+\bar{s}\geq 0\;⟹\;-2\delta -s-\bar{s}\leq 0\;.$$

Finally, the damping sector with the angle θ, $0\leq \theta <\frac{\pi }{2}$, corresponds to the constraint

(A5) ℜ{s}·tanθ≤−ℑ{s}⟹ℜ{s}·sinθ≤−ℑ{s}·cosθ.

Due to the negativity of both sides, squaring the second inequality in (A5) yields

(A6) $${ℜ}^{2}\left\{s\right\}\cdot \sin^{2}\theta \geq {ℑ}^{2}\left\{s\right\}\cdot \cos^{2}\theta \;\mathrm{with}\;ℜ\left\{s\right\}\cdot \sin\theta \leq 0\;,$$

which is equivalent to

(A7) $${\left(\frac{s+\bar{s}}{2}\right)}^{2}\cdot \sin^{2}\theta \geq {\left(\frac{s-\bar{s}}{2j}\right)}^{2}\cdot \cos^{2}\theta \;\mathrm{with}\;\left(\frac{s+\bar{s}}{2}\right)\cdot \sin\theta \leq 0$$

with $j^{2}=-1$ and, hence, also

(A8) $${\left(s+\bar{s}\right)}^{2}\cdot \sin^{2}\theta +{\left(s-\bar{s}\right)}^{2}\cdot \cos^{2}\theta \geq 0\;\mathrm{with}\;\left(s+\bar{s}\right)\cdot \sin\theta \leq 0\;.$$

Combining both scalar inequalities into a joint matrix inequality with the constraints of negative definiteness yields (by accounting for Sylvester’s criterion)

(A9) $$\left[\begin{array}{cc} \left(s+\bar{s}\right)\cdot \sin\theta & \left(s-\bar{s}\right)\cdot \cos\theta \\ -\left(s-\bar{s}\right)\cdot \cos\theta & \left(s+\bar{s}\right)\cdot \sin\theta \end{array}\right]=s\cdot \left[\begin{array}{cc} \sin\theta & \cos\theta \\ -\cos\theta & \sin\theta \end{array}\right]+\bar{s}\cdot \left[\begin{array}{cc} \sin\theta & -\cos\theta \\ \cos\theta & \sin\theta \end{array}\right]⪯0\;.$$

Now, a combination of all inequalities (A2), (A4), and (A9) in the form

(A10) $$\underset{D_{0}}{\underset{︸}{\left[\begin{array}{cccc} 2\gamma & 0 & 0 & 0\\ 0 & -2\delta & 0 & 0\\ 0 & 0 & 0 & 0\\ 0 & 0 & 0 & 0 \end{array}\right]}}+s\cdot \underset{D_{1}}{\underset{︸}{\left[\begin{array}{cccc} 1 & 0 & 0 & 0\\ 0 & -1 & 0 & 0\\ 0 & 0 & \sin\theta & \cos\theta \\ 0 & 0 & -\cos\theta & \sin\theta \end{array}\right]}}+\bar{s}\cdot D_{1}^{T}⪯0$$

completes the derivation of the matrices listed in (24).

**Remark** **A1.**
*In contrast to the following Appendix B, the inequalities above are formulated as semi-definiteness constraints to allow for the case θ=0 considered in the application scenario.*

## Appendix B. Generalization of (13) toward the Consideration of the Eigenvalue Region Constraints of the Output Feedback Controller

To account for the consideration of eigenvalue constraints in the form

(A11) $$F_{\Gamma ,C}\left(s\right):=D_{0,C}+s\cdot D_{1,C}+\bar{s}\cdot D_{1,C}^{T}≺0$$

with the given matrices $D_{0,C}$ and $D_{1,C}$ in the sense of a generalization of Corollary 1, the inequality constraint (11) is first replaced by

(A12) $$D_{0,C}⊗P+D_{1,C}⊗\left({\left(A_{v}-B_{v}K_{y}C_{y}^{+}\bar{C}\right)}^{T}\cdot P\right)+D_{1,C}^{T}⊗\left(P\cdot \left(A_{v}-B_{v}K_{y}C_{y}^{+}\bar{C}\right)\right)≺0$$

for each polytope vertex *v*. A multiplication of this inequality from the left and right with the symmetric block diagonal matrix $\mathrm{blkdiag}\left(Q,\ldots ,Q\right):=\mathrm{blkdiag}\left(P^{-1},\ldots ,P^{-1}\right)≻0$ of appropriate dimension yields

(A13) $$D_{0,C}⊗Q+D_{1,C}⊗\left(Q\cdot {\left(A_{v}-B_{v}K_{y}C_{y}^{+}\bar{C}\right)}^{T}\right)+D_{1,C}^{T}⊗\left(\left(A_{v}-B_{v}K_{y}C_{y}^{+}\bar{C}\right)\cdot Q\right)≺0$$

and

(A14) $$D_{0,C}⊗Q+D_{1,C}⊗\left(QA_{v}^{T}-Q{\left(C_{y}^{+}\bar{C}\right)}^{T}K_{y}^{T}{B_{v}}^{T}\right)+D_{1,C}^{T}⊗\left(A_{v}Q-B_{v}K_{y}C_{y}^{+}\bar{C}Q\right)≺0\;,$$

which by applying the variable substitution

(A15) $$NC_{y}^{+}\bar{C}=K_{y}MC_{y}^{+}\bar{C}=K_{y}C_{y}^{+}\bar{C}Q\;,$$

resulting from the equality constraint (12) turns into

(A16) $$D_{0,C}⊗Q+D_{1,C}⊗\left(QA_{v}^{T}-{\left(C_{y}^{+}\bar{C}\right)}^{T}N^{T}{B_{v}}^{T}\right)+D_{1,C}^{T}⊗\left(A_{v}Q-B_{v}NC_{y}^{+}\bar{C}\right)≺0\;.$$

**Remark** **A2.**
*Although (A16) is an obvious substitute for (13), it should be pointed out that the output feedback approach derived in this paper was designed on the assumption of the stabilizability of the plant by the considered system outputs; see the discussion of the control law (6) in Case 3. Imposing the additional constraint (A11) instead of purely demanding ℜ{s}<0 may make the design tasks infeasible due to the fact that a fully free, independent eigenvalue placement is typically impossible by means of a pure output feedback control.*
